# Supplementary material for: The use of mobile apps and fitness trackers to promote healthy behaviors during COVID-19: A cross-sectional survey
Source: PLOS Digit Health. 2022 Aug 18;1(8):e0000087. doi: 10.1371/journal.pdig.0000087 (PMC9931267; doi:10.1371/journal.pdig.0000087)
Supplement: S2 Appendix — (PDF) [file pdig.0000087.s002.pdf]

Appendix 2: Survey items

Survey categories	Questions
Demographic information	1. What is your gender?<br><br>2. What is your age? (in years)<br><br>3. What is the highest level of education you have completed?<br><br>4. In which country do you currently reside?
Current health status	5. Do you have a current medical condition for which you require regular medical care or medication?<br><br>6. Are you a cigarette smoker?<br><br>7. How many cigarettes do you typically smoke in a day?<br><br>8. On average, how many **standard drinks** do you have in a week?<br>9. (**A standard drink** contains 10 grams/0.35 ounces of alcohol, e.g.: 100ml of wine (12% alc/vol), 30ml of spirits (40%alc/vol), 280ml of standard strength beer (4.9% alc/vol)<br>10. If you have 6 standard drinks per week, enter 6.<br><br>11. In general, how many **minutes** of moderate to vigorous physical activity do you participate in each week?<br>12. (**Moderate to vigorous activity** is an activity which makes you breathe harder, such as walking, vacuuming, yoga.)<br>13. If you do 30 minutes per week, enter 30.<br><br>14. In general, how many servings of vegetables do you have in a day?<br>15. (**One serving** = 1/2 cup of cooked vegetables, 1/2cup beans, peas or lentils. Includes fresh, frozen and canned vegetables.)<br><br>16. In general, how many servings of fruits do you have in a day?<br>17. (**One serving** = 1 medium apple, 1 cup canned fruit or 1 cup chopped fresh fruit. Includes fresh, frozen and canned fruit.)
COVID-19 related apps	18. There are several mobile apps that were designed specifically for COVID-19 (e.g. contact tracing to reduce the spread). Are you using any COVID-19 related apps? If yes, please specify. You can select multiple options.<br><br>19. For what purposes are you using the COVID-19 related apps? You can select multiple options.
Technology use for healthy lifestyle and mental wellbeing	The following questions will examine your use of technology to support **healthy lifestyle and mental wellbeing**. Healthy lifestyle includes **any** of the following: staying active, eating healthily, sleeping better, reducing/stopping smoking and alcohol drinking, and managing mental

	wellbeing. We are **not** focusing on chronic disease management (e.g. monitor blood glucose, medication reminder).
Mobile apps	The next section will ask you about your mobile app use. Feel free to check your mobile phone while answering the questions.<br><br>20. Are you using mobile apps to support a healthy lifestyle and/or mental wellbeing (e.g. Apple Health, Fitbit, Facebook-to attend an online exercise class)?<br><br>21. **Before** the outbreak of COVID-19, did you use mobile apps **consistently** (e.g. use an app more than 5 times) to support a healthy lifestyle and/or mental wellbeing?<br><br>22. **Before** the outbreak of COVID-19, which mobile apps have you used **consistently** (e.g. more than 5 times) to support a healthy lifestyle and/or mental wellbeing? You can select multiple options.<br><br>23. Since the outbreak of COVID-19, how has your use of mobile apps to support a healthy lifestyle and/or mental wellbeing changed?<br><br>24. Since the outbreak of COVID-19, did you start using a **new** mobile app (that you have never used before) to support a healthy lifestyle and/or mental wellbeing?<br><br>25. Since the outbreak of COVID-19, which **new** mobile apps have you started to use to support a healthy lifestyle and/or mental wellbeing? You can select multiple options.<br><br>26. Specifically, for what health purpose do you use mobile apps during the COVID-19 pandemic? You can select multiple options.<br><br>27. During the COVID-19 pandemic, how have you used mobile apps for your physical activity? You can select multiple options.<br><br>28. How much do you agree with the following statements? During the COVID-19 pandemic, using mobile apps has helped me:<br><br>- Incorporate more activity in my day<br>- Eat healthier<br>- Sleep better<br>- Reduce/quit drinking alcohol<br>- Reduce/quit smoking<br>- Manage my mental wellbeing

	29. Do you have any other comments regarding how mobile apps have helped you maintain a healthy lifestyle and/or mental wellbeing during the COVID-19 pandemic? (optional)
Fitness trackers	30. Are you using a fitness tracker (e.g. Fitbit, Apple Watch, Garmin)?<br><br>31. Did you use fitness trackers to support a healthy lifestyle and/or mental wellbeing **before** the outbreak of COVID-19?<br><br>32. Which fitness trackers did you use to support a healthy lifestyle and/or mental wellbeing **before** the outbreak ofCOVID-19? You can select multiple options.<br><br>33. How long (in months) have you used fitness trackers for your health?<br><br>34. Since the outbreak of COVID-19, did you start using a **new** fitness tracker (that you have not used before) to support a healthy lifestyle and/or mental wellbeing?<br><br>35. Since the outbreak of COVID-19, which **new** fitness trackers have you started to use to support a healthy lifestyle and/or mental wellbeing? You can select multiple options.<br><br>36. During the COVID-19 pandemic, how have you used fitness trackers to support a healthy lifestyle and/or mental wellbeing? You can select multiple options.<br><br>37. How much do you agree with the following statements?<br>38. During the COVID-19 pandemic, using fitness trackers has helped me:<br>- Incorporate more activity in my day<br>- Eat healthier<br>- Sleep better<br>- Reduce/quit drinking alcohol<br>- Reduce/quit smoking<br>- Manage my mental wellbeing<br><br>39. Do you have any other comments regarding how fitness trackers have helped you maintain a healthy lifestyle and/or mental wellbeing during the COVID-19 pandemic? (optional)
Additional survey information	40. Is there anything else you'd like to tell us about your use of technology to support a healthy lifestyle and mental wellbeing that hasn't been captured in the survey? (optional)

MACQUARIE University

Default Question Block

Participant Information and Consent Form

Name of Project: Use of digital technologies to support health and wellbeing during the outbreak of COVID-19

Introduction
You are invited to take part in a survey which looks at how you use technologies to support your health and wellbeing during the outbreak of COVID-19. Before you decide whether or not you wish to take part, it is important for you to understand why the research is being done and what it will involve. Please take the time to read the following information carefully and discuss it with others if you wish.

Why are we doing the study?
The purpose of this research is to understand how people use digital technologies (e.g. mobile apps, fitness trackers, social media) to maintain healthy behaviour and mental wellbeing during the COVID-19 pandemic. This information may help us to design technologies and to be better prepared
to support people in achieving and maintaining optimal health and wellbeing during times of crisis.

This study is being conducted as part of the Doctor of Philosophy program at Macquarie University for Ms Huong Ly Tong (huong-ly.tong@hdr.mq.edu.au) under the supervision of Dr Liliana Laranjo (liliana.laranjo@mq.edu.au), and Dr Juan Quiroz (juan.quiroz@mq.edu.au from the Australian Institute of Health Innovation.

What does participation in this study involve?
You will fill out a online survey, which collects information on your age, gender, current health status, and usage of digital technologies. It will take you 5 to 10 minutes to complete the survey.

How will your information be used?
Your responses to the surveys are stored by Qualtrics in California, USA. Upon the end of data collection, the survey answers will be downloaded and then deleted from Qualtrics. The data will be stored on a password-protected drive on the cloud-based file-hosting service CloudStor.
Your data might be presented in an aggregate format in scientific journals and conferences.
Upon study completion, a non-identifiable dataset containing the survey response will be made publicly available in the Open Science Framework platform (https://osf.io/). No personal identifying information will be included. These data are shared to promote transparency and reproducibility in research, and can only be used for research purposes.

What are the benefits for taking part in the study?
This study may allow participants to reflect upon their use of digital technologies to support their health during the outbreak of COVID-19. This study may also play an important role in designing technologies to help us be better prepared in future health emergencies.

Are there any risks to being part of the study?
The risks involved in the study are low and include the time burden associated with your participation. You might experience some discomfort thinking about your health and wellbeing during the outbreak of COVID-19.

What are the rights of participants in the study?
Your participation is entirely voluntary. You can edit your responses anytime while filling out the survey. After submitting your response, you will not be able to modify your answers. You are free to say no to participating, or leave

the survey at any time, without having to give a reason and without any consequences. If you withdraw from the study, we will not collect additional personal information from you, although personal information already collected will be retained to ensure that the results of the research project can be measured properly. You should be aware that data collected up to the time you withdraw will form part of the research project results.

All information is kept confidential. We will keep the study data for a minimum of 5 years from the most recent publication date and store the data securely. A summary of the results of the data can be made available to you on request, by emailing Huong Ly Tong at huong-ly.tong@hdr.mq.edu.au. Alternatively, a link to the study publication will also be sent to your email upon completion, if you choose to leave your email address at the end of the survey.

Who should I contact for more information?
You can contact Ms Huong Ly Tong via email at huong-ly.tong@hdr.mq.edu.au.

Who should I contact if I have concerns or complaints about the study?
The Macquarie University Human Research Ethics Committee has approved the ethical aspects of this study (approval number: 52020674017063). If you have any complaints or reservations about any ethical aspect of your participation in this research, you may contact the Committee through the Director, Research Ethics & Integrity (Ph +61 2 9850 7854; email ethics@mq.edu.au). Any complaint you make will be treated in confidence and investigated, and you will be informed of the outcome.

CONSENT FORM
Before taking part in the study, I confirm that:
1. I have read and understood the Participant Information Sheet for the study.
2. I have had sufficient time to consider whether or not to take part in the study.
3. I am 18 years or older.

I also understand that my participation is voluntary and that I am free to withdraw, by leaving this survey at any time, without giving any reason.

Do you consent to take part in this study?

○ Yes
○ No

Demographic information

What is your gender?

○ Male
○ Female

- ○ Other
- ○ Prefer not to say

What is your age? (in years)

[ ]

What is the highest level of education you have completed?

- ○ Primary School
- ○ High School
- ○ TAFE/certificate/diploma/apprenticeship
- ○ Undergraduate bachelor degree
- ○ Postgraduate degree

Country

In which country do you currently reside?

[▾]

Current health status

Do you have a current medical condition for which you require regular medical care or medication?

- ○ [ ] Yes. Please specify.

○ No

Are you a cigarette smoker?

○ Yes
○ No

How many cigarettes do you typically smoke in a day?

On average, how many **standard drinks** do you have in a week?
(**A standard drink** contains 10 grams/0.35 ounces of alcohol, e.g.: 100ml of wine (12% alc/vol), 30ml of spirits (40% alc/vol), 280ml of standard strength beer (4.9% alc/vol)
If you have 6 standard drinks per week, enter 6.

In general, how many **minutes** of moderate to vigorous physical activity do you participate in each week?
(**Moderate to vigorous activity** is an activity which makes you breathe harder, such as walking, vacuuming, yoga.)
If you do 30 minutes per week, enter 30.

In general, how many servings of vegetables do you have in a day?
(**One serving** = 1/2 cup of cooked vegetables, 1/2 cup beans, peas or lentils. Includes fresh, frozen and canned vegetables.)

In general, how many servings of fruits do you have in a day?
(**One serving** = 1 medium apple, 1 cup canned fruit or 1 cup chopped fresh fruit. Includes fresh, frozen and canned fruit.)

COVID-19 related apps

There are several mobile apps that were designed specifically for COVID-19 (e.g. contact tracing to reduce the spread). Are you using any COVID-19 related apps? If yes, please specify. You can select multiple options.

- ☐ No, I am not using any COVID-19 related apps.
- ☐ COVIDSafe by Australian Department of Health
- ☐ Coronavirus Australia
- ☐ Healthlynked COVID-19 tracker
- ☐ NHS COVID-19
- ☐ Others – please specify (please put in app names, separated by a comma – e.g. App 1, App 2)

For what purposes are you using the COVID-19 related apps? You can select multiple options.

- ☐ To support contact tracing (i.e. to find and be notified if you have come into close contact with a COVID-19 case)
- ☐ To check symptoms of COVID-19
- ☐ To get information and updates about COVID-19
- ☐ Others - please specify ____________

TECHNOLOGY USE FOR HEALTHY LIFESTYLE AND MENTAL WELLBEING

The following questions will examine your use of technology to support **healthy lifestyle and mental wellbeing**. Healthy lifestyle includes **any** of the following: staying active, eating healthily, sleeping better, reducing/stopping smoking and alcohol drinking, and managing mental wellbeing. We are **not** focusing on chronic disease management (e.g. monitor blood glucose, medication reminder).

Mobile apps

The next section will ask you about your mobile app use. Feel free to check your mobile phone while answering the questions.

Are you using mobile apps to support a healthy lifestyle and/or mental wellbeing (e.g. Apple Health, Fitbit, Facebook- to attend an online exercise class)?

- ◯ Yes, I am using mobile apps for my health.
- ◯ No, I used some apps in the past, but I am not using one now.
- ◯ No, I have never used mobile apps for my health.

Before the outbreak of COVID-19, did you use mobile apps **consistently** (e.g. use an app more than 5 times) to support a healthy lifestyle and/or mental wellbeing?

- ◯ Yes
- ◯ No

Before the outbreak of COVID-19, which mobile apps have you used **consistently** (e.g. more than 5 times) to support a healthy lifestyle and/or mental wellbeing? You can select multiple options.

- ☐ Apple Health
- ☐ Samsung Health
- ☐ Fitbit
- ☐ Strava
- ☐ MyFitnessPal
- ☐ DrinkControl
- ☐ My QuitBuddy
- ☐ MyQuit Coach
- ☐ Headspace
- ☐ Happify
- ☐ Calm
- ☐ Facebook
- ☐ Twitter

- [ ] Instagram
- [ ] Reddit
- [ ] Snapchat
- [ ] Youtube (e.g. to follow exercise video)
- [ ] Zoom (e.g. to join a live exercise class)
- [ ] Houseparty
- [ ] Others (please put in app names, separate by a comma – e.g. App 1, App 2)

Since the outbreak of COVID-19, how has your use of mobile apps to support a healthy lifestyle and/or mental wellbeing changed?

- ○ I use mobile apps more.
- ○ I use mobile apps the same as before COVID-19.
- ○ I use mobile apps less.

Since the outbreak of COVID-19, did you start using a **new** mobile app (that you have never used before) to support a healthy lifestyle and/or mental wellbeing?

- ○ Yes
- ○ No

Since the outbreak of COVID-19, which **new** mobile apps have you started to use to support a healthy lifestyle and/or mental wellbeing? You can select multiple options.

- [ ] Apple Health
- [ ] Samsung Health
- [ ] Fitbit

- ☐ Strava
- ☐ MyFitnessPal
- ☐ DrinkControl
- ☐ My QuitBuddy
- ☐ MyQuit Coach
- ☐ Headspace
- ☐ Happify
- ☐ Calm
- ☐ Facebook
- ☐ Twitter
- ☐ Instagram
- ☐ Reddit
- ☐ Snapchat
- ☐ Youtube (e.g. to follow exercise video)
- ☐ Zoom (e.g. to join a live exercise class)
- ☐ Houseparty
- ☐ Others – Please specify (please put in app names, separated by a comma – e.g. App 1, App 2)

Specifically, for what health purpose do you use mobile apps during the COVID-19 pandemic? You can select multiple options.

- ☐ To stay active
- ☐ To eat healthily
- ☐ To sleep better
- ☐ To reduce/quit smoking
- ☐ To reduce/quit drinking alcohol
- ☐ To connect with other people
- ☐ To manage my mental health
- ☐ Others – please specify

During the COVID-19 pandemic, how have you used mobile apps for your physical activity? You can select multiple options.

- ☐ To track how much I have walked/run
- ☐ To join a live class (e.g. Zoom, Facebook live)
- ☐ To follow an exercise video (e.g. follow a yoga video on youtube)
- ☐ To compete with my family/friends
- ☐ To share my activity and progress with others
- ☐ [ ] Others – please specify

How much do you agree with the following statements? During the COVID-19 pandemic, using mobile apps has helped me:

	Strongly agree	Somewhat agree	Neither agree nor disagree	Somewhat disagree	Strongly disagree	Not applicable
Incorporate more activity in my day	○	○	○	○	○	○
Eat healthier	○	○	○	○	○	○
Sleep better	○	○	○	○	○	○
Reduce/quit drinking alcohol	○	○	○	○	○	○
Reduce/quit smoking	○	○	○	○	○	○
Manage my mental wellbeing	○	○	○	○	○	○

Do you have any other comments regarding how mobile apps have helped you maintain a healthy lifestyle and/or mental wellbeing during the COVID-19 pandemic? (optional)

Fitness trackers

Are you using a fitness tracker (e.g. Fitbit, Apple Watch, Garmin)?

- ○ Yes, I am using one now.
- ○ No, I used one in the past but stopped.
- ○ No, I have never used fitness trackers.

Did you use fitness trackers to support a healthy lifestyle and/or mental wellbeing **before** the outbreak of COVID-19?

- ○ Yes
- ○ No

Which fitness trackers did you use to support a healthy lifestyle and/or mental wellbeing **before** the outbreak of COVID-19? You can select multiple options.

- ☐ Apple watch
- ☐ Fitbit
- ☐ Garmin
- ☐ Others - please specify

How long (in months) have you used fitness trackers for your health?

[ ]

Since the outbreak of COVID-19, did you start using a **new** fitness tracker (that you have not used before) to support a healthy lifestyle and/or mental wellbeing?

- ○ Yes
- ○ No

Since the outbreak of COVID-19, which **new** fitness trackers have you started to use to support a healthy lifestyle and/or mental wellbeing? You can select multiple options.

- ☐ Apple watch
- ☐ Fitbit
- ☐ Garmin
- ☐ [ ] Others - please specify

During the COVID-19 pandemic, how have you used fitness trackers to support a healthy lifestyle and/or mental wellbeing? You can select multiple options.

- ☐ To track how much I have walked/run
- ☐ To track my heart rate
- ☐ To track how many calories I have burned
- ☐ To receive reminders to move

- [ ] To track my sleep
- [ ] To practice mindfulness
- [ ] To compete with family/friends
- [ ] Others – please specify

How much do you agree with the following statements? During the COVID-19 pandemic, using fitness trackers has helped me:

	Strongly agree	Somewhat agree	Neither agree nor disagree	Somewhat disagree	Strongly disagree	Not applicable
Incorporate more activity in my day	○	○	○	○	○	○
Eat healthier	○	○	○	○	○	○
Sleep better	○	○	○	○	○	○
Reduce/quit drinking alcohol	○	○	○	○	○	○
Reduce/quit smoking	○	○	○	○	○	○
Manage my mental wellbeing	○	○	○	○	○	○

Do you have any other comments regarding how fitness trackers have helped you maintain a healthy lifestyle and/or mental wellbeing during the COVID-19 pandemic? (optional)

Additional survey info

Is there anything else you'd like to tell us about your use of technology to support a healthy lifestyle and mental wellbeing that hasn't been captured in the survey? (optional)

Confirmation

You can go back and edit your responses if you like. If you are ready, please submit your responses. After submission, you will not be able to modify your answers.

Powered by Qualtrics
