# Supplementary material for: The use of mobile apps and fitness trackers to promote healthy behaviors during COVID-19: A cross-sectional survey
Source: PLOS Digit Health. 2022 Aug 18;1(8):e0000087. doi: 10.1371/journal.pdig.0000087 (PMC9931267; doi:10.1371/journal.pdig.0000087)
Supplement: S3 Appendix — (DOCX) [file pdig.0000087.s003.docx]

| Country | Number of responses | Percentage |
| --- | --- | --- |
| Afghanistan | 1 | 0.2% |
| Australia | 382 | 69.6% |
| Brunei | 1 | 0.2% |
| Canada | 7 | 1.3% |
| Chile | 1 | 0.2% |
| Croatia | 1 | 0.2% |
| Denmark | 2 | 0.4% |
| Germany | 4 | 0.7% |
| Greece | 1 | 0.2% |
| Hong Kong | 1 | 0.2% |
| India | 5 | 0.9% |
| Israel | 2 | 0.4% |
| Italy | 1 | 0.2% |
| Japan | 3 | 0.5% |
| Lithuania | 1 | 0.2% |
| Malaysia | 2 | 0.4% |
| Mexico | 1 | 0.2% |
| Netherlands | 3 | 0.5% |
| Pakistan | 1 | 0.2% |
| Philippines | 3 | 0.5% |
| Portugal | 4 | 0.7% |
| Singapore | 3 | 0.5% |
| South Africa | 1 | 0.2% |
| South Korea | 1 | 0.2% |
| Sweden | 3 | 0.5% |
| Switzerland | 2 | 0.4% |
| Turkey | 1 | 0.2% |
| United Arab Emirates | 1 | 0.2% |
| UK | 23 | 4.2% |
| USA | 52 | 9.5% |
| Vietnam | 35 | 6.4% |
| Total | 549 |  |

**Appendix 3: Country of residence breakdown by the number of responses and %**
