# Supplementary material for: The use of mobile apps and fitness trackers to promote healthy behaviors during COVID-19: A cross-sectional survey
Source: PLOS Digit Health. 2022 Aug 18;1(8):e0000087. doi: 10.1371/journal.pdig.0000087 (PMC9931267; doi:10.1371/journal.pdig.0000087)
Supplement: S4 Appendix — (DOCX) [file pdig.0000087.s004.docx]

**Appendix 4: Subgroup analyses**

**Table A: Gender differences in the use of mobile apps for healthy behaviors and mental wellbeing**

| n/N (%) | Women | Men | p-values |
| --- | --- | --- | --- |
| Current app usage |  |  | .004 |
| Yes | 249/389 (64) | 51/109 (46.8) |  |
| No | 140/389 (36) | 58/109 (53.2) |  |
| Use app to stay active |  |  | .02 |
| Yes | 203/423 (48) | 44/120 (36.7) |  |
| No | 220/423 (52) | 76/120 (63.3) |  |
| Use app to connect with other people |  |  | .004 |
| Yes | 96/423 (22.7) | 11/120 (9.2) |  |
| No | 327/423 (77.3) | 109/120 (90.8) |  |
| Found mobile app helpful for managing mental wellbeing |  |  | .04 |
| Yes | 141/185 (76.2) | 24/38 (63.2) |  |
| No | 44/185 (23.8) | 14/38 (36.8) |  |

**Table B: Differences in the use of COVID-19 apps by age group and status of mobile app usage (i.e., whether people were using mobile apps for health-related purposes)**

| n/N (%) | Use of COVID-19 apps | | p-values |
| --- | --- | --- | --- |
|  | Yes | No |  |
| Age group |  |  | <.001 |
| 18—44 | 187/406 (46.1) | 219/406 (53.9) |  |
| 45—60 | 57/99 (57.6) | 42/99 (42.4) |  |
| 60+ | 35/47 (74.5) | 12/47 (25.5) |  |
| Use mobile apps for health |  |  | .034 |
| Yes – current users | 152/302 (50.3) | 150/302 (49.7) |  |
| Yes – past users | 49/103 (47.6) | 54/103 (52.4) |  |
| No - never-users | 35/99 (35.3) | 64/99 (64.7) |  |
