# Supplementary material for: The use of mobile apps and fitness trackers to promote healthy behaviors during COVID-19: A cross-sectional survey
Source: PLOS Digit Health. 2022 Aug 18;1(8):e0000087. doi: 10.1371/journal.pdig.0000087 (PMC9931267; doi:10.1371/journal.pdig.0000087)
Supplement: S5 Appendix — (DOCX) [file pdig.0000087.s005.docx]

**Appendix 5: Sensitivity analyses in the Australia sub-sample**

**Table A: Adjusted odds ratios (OR) and 95% confidence intervals (CI) for the associations between 1) adherence to aerobic physical activity guideline and use of mobile apps or fitness trackers; 2) adherence to fruit and vegetable consumption guideline and use of mobile apps in the Australia sub-sample (n=377)**

| **Variables** | **Odds ratio (95% CI) of adherence to aerobic physical activity guideline^a^** | **p-values** | **Odds ratio (95% CI) of adherence to fruit and vegetable guideline^b^** | **p-values** |
| --- | --- | --- | --- | --- |
| **Age** | 1.03 (1.01, 1.05) | *.004* | 1.01 (0.99, 1.03) | .20 |
| **Gender** |  |  |  |  |
| **Female** | 1 (reference level) |  | 1 (reference level) |  |
| **Male** | 1.76 (0.93, 3.37) | .08 | 0.84 (0.45, 1.57) | .59 |
| **Education** |  |  |  |  |
| **High school** | 0.86 (0.24, 2.93) | .81 | 0.36 (0.09, 1.16) | .10 |
| **Vocation training** | 0.46 (0.06, 3.04) | .42 | 2.10 (0.25, 43.81) | .53 |
| **Undergraduate degree** | 0.89 (0.49, 1.61) | .69 | 0.43 (0.23, 0.77) | *.005* |
| **Postgraduate degree** | 1 (reference level) |  | 1 (reference level) |  |
| **Current medical condition** |  |  |  |  |
| **Yes** | 0.76 (0.44, 1.31) | .32 | 0.97 (0.56, 1.66) | .90 |
| **No** | 1 (reference level) |  | 1 (reference level) |  |
| **Current app or tracker usage^c^** |  |  |  |  |
| **Yes** | 1.63 (0.79, 3.43) | .19 | 1.08 (0.52, 2.27) | .83 |
| **No** | 1 (reference level) |  | 1 (reference level) |  |
| **Whether an app or tracker was used pre-COVID^c^** |  |  |  |  |
| **Yes** | 2.82 (1.45, 5.60) | *.003* | 1.29 (0.70, 2.39) | .42 |
| **No** | 1 (reference level) |  | 1 (reference level) |  |
| **Whether a new app or tracker was used since COVID^c^** |  |  |  |  |
| **Yes** | 1.98 (1.15, 3.42) | *.01* | 1.39 (0.81, 2.41) | .24 |
| **No** | 1 (reference level) |  | 1 (reference level) |  |

^a^Participants were considered to have adhered to aerobic physical activity guideline if they self-reported doing at least 150 minutes of moderate to vigorous physical activity in a week; ^b^Participants were considered to have adhered to fruit and vegetable consumption guideline if they self-reported having at least 5 servings of fruits and vegetables in a day; ^c^The model exploring the link between technologies and fruit and vegetable consumption only considered app usage, not fitness trackers.
