# Supplementary material for: The use of mobile apps and fitness trackers to promote healthy behaviors during COVID-19: A cross-sectional survey
Source: PLOS Digit Health. 2022 Aug 18;1(8):e0000087. doi: 10.1371/journal.pdig.0000087 (PMC9931267; doi:10.1371/journal.pdig.0000087)
Supplement: S6 Appendix — (DOCX) [file pdig.0000087.s006.docx]

**Appendix 6: Demographic information of participants who responded to open-ended questions**

**Table A**: Demographic information of participants who responded to the open-ended question “Do you have any other comments regarding how mobile apps have helped you maintain a healthy lifestyle and/or mental wellbeing during the COVID-19 pandemic? (N = 110)

| **Demographic characteristics** | **N^a^** | **Percentage (%) or Mean (SD)** |
| --- | --- | --- |
| **Age (years)**; range: 18 – 80 |  | 40 (14.8) |
| 18 - 29 | 33 | 30% |
| 30 - 39 | 33 | 30% |
| 40 - 49 | 17 | 15.5% |
| 50+^b^ | 27 | 24.5% |
| **Gender** |  |  |
| Female | 94 | 85.5% |
| Male | 15 | 13.6% |
| Other | 1 | 0.9% |
| Prefer not to say | 0 | 0% |
| **Education level** |  |  |
| Primary School | 0 | 0% |
| High School | 8 | 7.3% |
| Vocational training | 2 | 1.8% |
| Undergraduate bachelor’s degree | 26 | 23.6% |
| Postgraduate degree | 74 | 67.3% |
| **Country of residence** |  |  |
| Australia | 81 | 73.6% |
| USA | 14 | 12.7% |
| Vietnam | 5 | 4.5% |
| Others | 10 | 9.1% |

**Table B**: Demographic information of participants who responded to the open-ended question “Do you have any other comments regarding how fitness trackers have helped you maintain a healthy lifestyle and/or mental wellbeing during the COVID-19 pandemic? (N = 32)

| **Demographic characteristics** | **N^a^** | **Percentage (%) or Mean (SD)** |
| --- | --- | --- |
| **Age (years)**; range: 24-77 |  | 40.7 (13.6) |
| 18 - 29 | 6 | 18.8% |
| 30 - 39 | 16 | 50% |
| 40 - 49 | 4 | 12.5% |
| 50+^b^ | 6 | 18.8% |
| **Gender** |  |  |
| Female | 25 | 78.1% |
| Male | 6 | 18.8% |
| Other | 1 | 3.1% |
| Prefer not to say | 0 | 0% |
| **Education level** |  |  |
| Primary School | 0 | 0% |
| High School | 0 | 0% |
| Vocational training | 1 | 3.1% |
| Undergraduate bachelor’s degree | 4 | 12.5% |
| Postgraduate degree | 27 | 84.4% |
| **Country of residence** |  |  |
| Australia | 25 | 78.1% |
| Canada | 3 | 9.4% |
| USA | 1 | 3.1% |
| Vietnam | 3 | 9.4% |

**Table C**: Demographic information of participants who responded to the open-ended question “Is there anything else you’d like to tell us about your use of technology to support a healthy lifestyle and mental wellbeing that hasn’t been captured in the survey?” (N = 102)

| **Demographic characteristics** | **N^a^** | **Percentage (%) or Mean (SD)** |
| --- | --- | --- |
| **Age (years)**; range: 19-80 |  | 41.6 (14.5) |
| 18 - 29 | 22 | 21.6% |
| 30 - 39 | 34 | 33.3% |
| 40 - 49 | 16 | 15.7% |
| 50+^b^ | 30 | 29.4% |
| **Gender** |  |  |
| Female | 80 | 78.4% |
| Male | 19 | 18.6% |
| Other | 2 | 2% |
| Prefer not to say | 1 | 1% |
| **Education level** |  |  |
| Primary School | 0 | 0% |
| High School | 3 | 2.9% |
| Vocational training | 4 | 3.9% |
| Undergraduate bachelor’s degree | 21 | 20.6% |
| Postgraduate degree | 74 | 72.5% |
| **Country of residence** |  |  |
| Australia | 76 | 74.5% |
| UK | 4 | 3.9% |
| USA | 7 | 6.9% |
| Vietnam | 8 | 7.8% |
| Others | 7 | 6.9% |
