# Supplementary material for: The use of mobile apps and fitness trackers to promote healthy behaviors during COVID-19: A cross-sectional survey
Source: PLOS Digit Health. 2022 Aug 18;1(8):e0000087. doi: 10.1371/journal.pdig.0000087 (PMC9931267; doi:10.1371/journal.pdig.0000087)
Supplement: Attachment — Submitted filename: 2022-06-06_Tong-response-to-reviewers.docx [file pdig.0000087.s007.docx]

Response to reviewers

**Manuscript: PDIG-D-21-00140 The use of mobile apps and fitness trackers to promote healthy behaviors during COVID-19: A cross-sectional survey**

**Academic Editor’s comment**: Please address the comments of the reviewers attached below. Several reviewers raised concerns regarding the testing and validation of the survey, lack of generalisability due to self-selection biases and the international nature of the survey, especially in the context of differences in Covid-19-related measures and health behaviour recommendations.

**Response:** We thank the Editor for considering our manuscript, and the reviewers for their valuable comments. We have addressed each comment below and revised the manuscript where needed. The colored text highlights the revision, and references to line number are to the track changes version of the manuscript.

In summary:

1. We responded to “the concerns regarding the testing and validation of the survey” in Reviewer 1 comment 1, and Reviewer 3 comment 1.
2. We responded to “the lack of generalisability due to self-selection biases and the international nature of the survey, especially in the context of differences in Covid-19-related measures and health behaviour recommendations.” in Reviewer 2 comment 1, Reviewer 2 comment 2, and Reviewer 3 comment 4.

**Journal Requirements**:

1. Please update your Competing Interests statement. If you have no competing interests to declare, please state: “The authors have declared that no competing interests exist.”

Response: Revised as requested (line 523).

| **Reviewers’ comments** | **Responses** |
| --- | --- |
| Reviewer 1: This is an interesting research.  I have highlighted a few comments to improve the manuscript. | Thank you. |
| 1. Was the survey items/questions validated? | The reviewer raised a good point. We have provided more details on the survey development:  “Survey development and measures  Existing COVID-19 surveys [44-46] were reviewed to inform the wording and structure of the present survey. Subsequently, a draft survey was prepared and reviewed independently in three rounds to establish face validity. Specifically, in round one, a draft survey was prepared by the first author and reviewed by a clinician and a computer science expert, with revisions made accordingly. In round two, the survey was sent out to three experts in digital health and behavioral research for feedback, and revised accordingly. Finally, the revision made in round two was reviewed again by a clinician prior to being finalized.” (lines 159-166)  We agree with the reviewer that validating survey items is important, and the fact that we only established face validity is a limitation of the study. We have discussed this in the Limitations section:  “While face validity was established through multiple co-authors independently reviewing the survey draft, the survey questions were not formally assessed for criterion or content validity, and the survey was not pilot tested.” (lines 473-476) |
| 2. Line 146: What is the rationale for the study duration? | We recruited participants until we achieved the targeted sample size of at least 500 people. This targeted sample size is based on published heuristics for sampling in behavioral research (Roscoe, 1975).  Roscoe JT. Fundamental research statistics for the behavioral sciences New York: Holt, Rinehart and Winston; 1975. ISBN: 0030919347.  We provided this detail in Appendix 1: STROBE checklist. We agreed with the reviewer that this is important to specify, and have amended the manuscript as followed:  “We followed published heuristics for sampling for behavioral research and aimed to recruit at least 500 participants into the study [38]. The survey was open from start of June to end of September 2020 to achieve the targeted sample size.” (lines 147-149) |
| 3. Why was snowball sampling explicitly used? | We thank the reviewer for pointing this out. We used the word “snowballing techniques” to describe the fact that in our social media advertisements, we asked participants to share the study information with their networks, in order to expand geographical scope of the study.  We acknowledged that using the word “snowballing techniques” is inappropriate here, as we did not apply the traditional snowballing sampling (i.e., participants nominated other potential participants for the study).  We have revised the manuscript to remove mention of snowballing and clarified our recruitment method:  “In our social media advertisements, we also asked people to share the study with their networks (e.g., re-tweet on Twitter), in order to expand the geographical scope of the study. ” (lines 140-143) |
| 4. Line 153: specify the period (date) rather than "during this time." | Revised as suggested:  “It is worth noting that during June – September 2020, a few countries had started to ease lifestyle restrictions (e.g., Australia, UK, Canada) [43]” (lines 156-158) |
| 5. Line 189: 5-point Likert scale? | Revised as suggested:  “These items were measured using a five-point Likert scale, ranging from strongly disagree to strongly agree.” (lines 200-202) |
| 6. Line 249: What is the frequency of Mobile App usage (e.g., once a week, daily, monthly)? | Unfortunately, we did not measure the frequency of mobile app usage. Instead, we asked if participants used mobile apps “consistently” to support their health, and we gave the example of “use an app more than 5 times” as consistent use. Previous research has demonstrated that user abandonment is a common issue in health technologies [PMID: 27745683], and industry report shows that 21% of all users abandoned an app after a single use (<https://uplandsoftware.com/localytics/resources/blog/21-percent-of-users-abandon-apps-after-one-use/> )  Such minimal use is unlikely to be associated with health impact, and thus, we asked whether people used mobile apps consistently.  We have clarified this in line 277:  “^a^People who used apps consistently (e.g., use an app more than 5 times) for health purposes in the past” |
| 7. Suggest putting survey items/questions in a table for easy understanding. | Per the reviewer’s suggestion, we have added the survey questions in a table in Appendix 2. |
| 8. Define the current, past, and never users categories (include references). Some studies use light or heavy users. | For the present study, we have provided the following definition as requested: Usage status during the pandemic was classified into three groups: current users, past users and never-users, based on existing literature [30, 31, 49]. The definition of usage status is provided in Box 1. (lines 197-199) Box 1: Classification based on technology usage during the pandemic* **Usage status** \| **Definition**; Current users \| People who were currently using mobile apps or fitness trackers for health purposes during the pandemic; Past users \| People who used mobile apps or fitness trackers for health purposes in the past, but were not currently using them during the pandemic; Never-users \| People who never used mobile apps or fitness trackers for health purposes **Note:** Classification based on [30, 31, 49]. [30, 31] classified participants into users and non-users. We modified this classification to include current users, and broke non-users into past and never-users based on [49] to provide more granularity in usage patterns. (lines 206-209) |
| 9. Did you identify the gamification features that users were interested in? | Thank you for your comment. We have modified the manuscript to provide examples of gamification features that the participants mentioned in open-ended responses:  “Many participants across different ages also valued gamification features of technologies (e.g., competition, exercise challenges, exercise role-playing games), which helped them to incorporate fitness into their life with an element of fun and enjoyment (Table 4, quotes 11 - 12).” (lines 384-387) |
| 10. Line 362-363: Suggest to detail the negative effects. | Revised as suggested:  “Qualitative findings complemented quantitative findings by showing while mobile devices helped maintain a sense of normalcy, there were potential negative effects of using technologies (e.g., stress and information overload from seeing COVID-19 information on social media, guilt when seeing low activity levels), which might have impacted users’ motivation and continued use of mobile devices.” (lines 400-405) |
| 11. Important to highlight the negative and positive effects on user motivation and adherence? | Thank you for your comment. The manuscript has been amended, as suggested:  “Qualitative findings complemented quantitative findings by showing while mobile devices helped maintain a sense of normalcy, there were potential negative effects of using technologies (e.g., stress and information overload from seeing COVID-19 information on social media, guilt when seeing low activity levels), which might have impacted users’ motivation and continued use of mobile devices.” (lines 400-405) |
| 12. Can gamification positively affect the adoption and post-adoption of the COVID-19 apps? | The reviewer raised an interesting hypothesis. In our qualitative analysis of open-ended responses, we did find that participants mentioned gamification as an engaging feature within mobile apps in general (lines 384-387).  We did not ask questions in the survey specifically about gamification features within COVID-19 apps. Thus, our study is not equipped to answer or speculate on this question. The impact of gamification on COVID-19 apps would be an interesting avenue for future research. |
| 13. Interested in knowing if these apps were theory-based and their relevance to the present of future study. | The reviewer raised an interesting point. In our study, we found that the most commonly used apps for health purposes were general and social media apps (e.g., Zoom, Facebook, Youtube), which are not theory-based (see Table 2,). We have amended the manuscript to better highlight this:  “The most popular apps used for health purposes during the pandemic were general and social media apps (e.g., Zoom, Facebook, Youtube), which were not purposedly built to promote health behavior change (Table 2).” (lines 271-274)  While previous research has shown that theory-based apps can have a greater impact on health behaviors, an interesting hypothesis for future research would be whether theory-based apps can also lead to higher engagement. |
| 14. The results can be expanded to compare with similar studies. An interesting study to consider is:  User Engagement and Abandonment of mHealth: A Cross-Sectional Survey (2022). In Healthcare (Vol. 10, No. 2, p. 221). MDPI.  Thank you. | Thank you. We have now cited this study:  “This is in line with previous research which suggests that personalization can increase user engagement with mobile devices [69].” (lines 497-499) |
| Reviewer 2**:**  1. The self-selection bias in this sample is so significant that I have to question the added value for research. But this is not limited to sociodemographic patterns. How valid are statements about subgroups of different usage behavior in a sample that seem to be strongly overrepresented by participants with higher health and fitness orientation? | 1. We agree with the reviewer that self-selection bias is a limitation of the study, and acknowledge this in the Strengths and limitations section (line 490).  2. An added value of our research is the mixed-methods design, which also include a qualitative component to better explore users’ perspectives. With this component, we were able to unveil the potential negative effects of technologies on health during the pandemic, and users’ preferences for technologies to adapt to changing circumstances. These findings have important implications for future research and technology design.  We discussed how the qualitative findings complemented quantitative findings in lines 400-406:  “Qualitative findings complemented quantitative findings by showing while mobile devices helped maintain a sense of normalcy, there were potential negative effects of using technologies (e.g., stress and information overload from seeing COVID-19 information on social media, guilt when seeing low activity levels), which might have impacted users’ motivation and continued use of mobile devices. Our participants highlighted the need for technologies to adapt to changing circumstances.”  Throughout the Discussion, we also discussed the implications of the qualitative findings regarding the need for adaptability in Impact on mobile devices on health behaviors section (lines 423-431), and Implications first paragraph (lines 498-503).  3. Finally, we also agree with the reviewer that our sample might have an overrepresentation of participants with higher health and fitness orientation (which is a concern the reviewer also raised in comment 4). Thus, we have modified the Abstract and Conclusion to better highlight this issue:  “Use of mobile apps and fitness trackers during the pandemic was associated with higher levels of physical activity, in a sample of educated and likely health-conscious individuals.” (lines 45-47)  “Our study found a positive impact of mobile apps and fitness trackers on physical activity during the pandemic, in a sample of likely health-conscious and technology-inclined individuals.” (lines 513-515)  We noted the implications of this limitation on the interpretation of findings at several places throughout the manuscript:  “The generalizability of our quantitative findings is limited, given our sample of highly educated individuals who might have been more health-conscious and had better access/more inclined to use technologies.” (Principal results, line 398-400)  “Our findings may be influenced by the large proportion of highly educated individuals who might be more health-conscious and have access to technologies more easily than other population groups. Previous research has described this phenomenon as the “digital divide” [70, 71], which can widen existing social inequalities. The benefits of mobile apps and devices would be limited if they can only reach high socioeconomic status groups. Thus, efforts must be made to bridge this gap in technology adoption, such as through increasing access, promoting collaborative and inclusive design, and improving digital literacy [70, 71].” (Implications, lines 505-511)  “As a large proportion of participants were women, and had high level of education, this might bias our findings and affect the generalizability to other population groups.” (Strengths and limitations, lines 483-485) |
| 2. Another substantial issue is the inclusion of participants from such diverse national backgrounds. Covid 19 incidences and distancing rules varied massively around the globe within the corresponding survey period. Amongst other reasons, this was obvious, at least because of the seasonal variation in pandemic events in the northern vs. southern hemisphere and completely different policy measures (e.g., USA vs. Australia). Therefore, I would strongly recommend limiting the analyses to the Australian subpopulation. | We agree with the reviewer that COVID-19 rules varied across the globe, and even within the different states in Australia. Thus, we have conducted a sensitivity analysis with only Australia-based participants, given the large proportion of this group in the sample. We opt to present both the planned analysis of the whole sample and the post-hoc sensitivity analysis in Australia-based participants to adhere to transparent reporting principles, and discuss the implications of our findings.  The manuscript has been amended as followed:   1. Methods, Data analysis: “Post-hoc sensitivity analyses were conducted to include only Australia-based participants, given the large proportion of this group in the sample.” (lin230-232es) 2. Results: “Given the large proportion of Australia-based participants in our sample, we conducted a sensitivity analysis with this subgroup (Appendix 5). The sensitivity analysis showed that current app or tracker usage was no longer statistically associated with meeting aerobic physical activity guidelines (OR = 1.63, 95% CI 0.79 to 3.43). Age, whether participants used an app or tracker before COVID-19, and whether participants started using a new app or tracker since COVID-19 were statistically associated with meeting aerobic physical activity guidelines. Mobile app usage was also not associated with meeting fruit and vegetable consumption guidelines in this subgroup (OR = 1.08, 95% CI 0.52 to 2.27).” (lines 324-331) 3. Discussion: “A sensitivity analysis including only Australia-based participants found that current mobile app or tracker usage was not associated with adherence to physical activity guidelines. It is possible that the smaller sample size made it difficult to detect the difference. Given the inconsistency between the primary and sensitivity analyses, the potential physical activity benefits associated with mobile devices observed in our findings should be interpreted with caution, and future research is needed to ascertain the potential impact of mobile devices on health behaviors.” (lines 416-422) |
| 3. Furthermore, I would recommend not to focus the analyses of so-called “subgroups” only on differential frequencies of app usage depending on sociodemographic characteristics. The authors should consider applying methods for exploring subgroups within the sample instead, e.g., cluster analysis or latent class analyses | We thank the reviewer for this comment. We agree with the reviewer that data-driven analyses such as cluster analysis or latent class analysis can be a valuable approach. In our study, we decided to conduct subgroup analysis of age and gender a priori, based on findings from previous cross-sectional surveys. We have amended the Methods to provide a justification for our decision:  “These subgroups were chosen based on the literature, as previous cross-sectional surveys have found that app usage might differ by age and gender [30, 35]. Specifically, Thomas et al found that COVID-19 app downloads appeared to increase with age, with the 65+ age group having the highest proportion of downloads [35]. Additionally, Parker et al also found that more women than men used digital platforms for their physical activity during the pandemic [30].” (lines 235-240)  We also amended the Introduction as followed:  “Despite rapid growth in the number of COVID-19 mobile apps, little is known about their adoption, with preliminary evidence suggesting that specific subgroups (e.g., older people) are more likely to adopt such apps [35].” (lines 111-114)  We also agree with the reviewer that subgroup analysis findings should be interpreted with caution. We have amended the manuscript in several places to highlight this:  Principal results (lines 396-398): “We note that these subgroup analyses based on age and gender are exploratory in nature and should be confirmed in future research.”  Differences in app usage between genders (lines 436-437): “It is worth noting that this gender difference is based on a subgroup analysis and is exploratory in nature.”  Adoption and usage of COVID-19 related apps (lines 467-469):” Furthermore, given the exploratory nature of this subgroup analysis, future research is needed to confirm potential age differences in COVID-19 app uptake.” |
| 4. I think using an app tracker before the pandemic is a strongly confounded proxy for interest in technology, as this may also indicate increased health and fitness orientation of the participants in particular. (For example, only 3% reported being smokers , with an average of 0.2 cigarettes a week!). | We agree with the reviewer that people who used an app or tracker before the pandemic were likely to have an interest in their health and fitness. We discussed the implications of these confounding relationship in the Discussion:  “Our results are consistent with existing literature showing that users of mobile apps and other digital technologies seem to be more active than non-users during the pandemic [29-31, 55]. Uniquely, by adjusting our model to variables related to ‘previous use of mobile devices before COVID’ and ‘adoption of new apps or trackers during the outbreak’, we found these were associated with adherence to physical activity guidelines. It is possible that the physical activity benefits observed in our study are influenced by an overrepresentation in our sample of health-conscious and tech-adopting people. Future research is needed to understand how mobile devices can extend its reach and benefit other groups beyond the typical highly motivated and ‘worried-well’ adopters [56].” (lines 408-416)  We agree with the reviewer that there is an overrepresentation of people who are highly educated and health-conscious in our sample.  We have modified the Abstract and Conclusion to better highlight this issue:  “Use of mobile apps and fitness trackers during the pandemic was associated with higher levels of physical activity, in a sample of educated and likely health-conscious individuals.” (lines 45-47)  “Our study found a positive impact of mobile apps and fitness trackers on physical activity during the pandemic, in a sample of likely health-conscious and technology-inclined individuals.” (lines 513-515)  We noted the implications of this limitation on the interpretation of findings at several places throughout the manuscript:  “The generalizability of our quantitative findings is limited, given our sample of highly educated individuals who might have been more health-conscious, and had better access and more inclined to use technologies.” (Principal results, lines 398-400)  “Our findings may be influenced by the large proportion of highly educated individuals who might be more health-conscious and have access to technologies more easily than other population groups. Previous research has described this phenomenon as the “digital divide” [70, 71], which can widen existing social inequalities. The benefits of mobile apps and devices would be limited if they can only reach high socioeconomic status groups. Thus, efforts must be made to bridge this gap in technology adoption, such as through increasing access, promoting collaborative and inclusive design, and improving digital literacy [70, 71].” (Implications, lines 505-511)  “As a large proportion of participants were women, and had high level of education, this might bias our findings and affect the generalizability to other population groups.” (Strengths and limitations, lines 483-485) |
| Reviewer 3: Thank you for this interesting manuscript describing technology usage and healthy behaviors during COVID-19. Please find below my comments: | Thank you. |
| Major comments:  1) I would like to know if you pilot-tested the survey. If not, could you please describe the methodology that you followed in order to create a validated survey? | We have provided more details on the survey development:  “Survey development and measures  Existing COVID-19 surveys [44-46] were reviewed to inform the wording and structure of the present survey. Subsequently, a draft survey was prepared and reviewed independently in three rounds to establish face validity. Specifically, in round one, a draft survey was prepared by the first author and reviewed by a clinician and a computer science expert, with revisions made accordingly. In round two, the survey was sent out to three experts in digital health and behavioral research for feedback, and revised accordingly. Finally, the revision made in round two was reviewed again by a clinician prior to being finalized.” (lines 159-166)  We agree with the reviewer that the lack of pilot testing is a study limitation, and we have discussed this in the Limitations section:  “While face validity was established through multiple co-authors independently reviewing the survey draft, the survey questions were not formally assessed for criterion or content validity, and the survey was not pilot tested.” (lines 473-476) |
| 2) "Diet was assessed by self-report of daily servings of vegetables and fruits."  Why did you assess only those two food categories? I would appreciate it if you could provide data on the rest of the food groups. | We agree with the reviewer that fruit and vegetables consumption is only a part of diet. We chose these categories as they were easy to understand and meaningful to a lay person. Additionally, we also wanted to keep the survey concise, in order to avoid drop-out (which is common in lengthy surveys).  We have modified the Methods section to emphasize this:  “Participants self-reported daily servings of vegetables and fruits.” (lines 182-183)  We agree that not collecting data on other food categories is a limitation of the study, and we cannot make wider claims regarding diet.  We have modified the Limitation section:  “We assessed the impact of technologies on only aerobic physical activity and the intake of fruits and vegetables. To enable a more comprehensive analysis on the link between technologies and physical activity and diet, future research should collect data on other types of activity (e.g., muscle strengthening exercises) and food groups (e.g., salt or sugar intake).” (lines 476-480) |
| 3) In Table 2, how did you define "more or less" in the usage of the app? | Changes in app usage during COVID-19 (e.g., more or less) were self-reported by the participants, in response to the survey item “Since the outbreak of COVID-19, how has your use of mobile apps to support a healthy lifestyle and/or mental wellbeing changed?” (Appendix 2).  To clarify that this is self-reported, we have edited the category name in Table 2 to “Self-reported changes in app usage during COVID-19” (page 17). |
| 4)"Mobile app usage was not associated with meeting fruit and vegetables consumption guidelines (OR = 0.97, 95% CI 0.53 to 1.76) (Table 3)"  Since you are including data from people coming from different countries and those ones have different guidelines, how did you end up with adherence or no to fruit and vegetable guidelines? | We agree with the reviewer that there are different guidelines amongst different countries.  Thus, we defined adherence to fruit and vegetables consumption based on the World Health Organization’s recommendation for adults.  We clarified this in the Methods section (lines 182-185):  “Participants were considered to have adhered the recommended intake of vegetables and fruits if they self-reported consuming at least five servings of vegetables and fruits in a day, based on the World Health Organization’s recommendation [48]” |
| 5) Could you maybe group the qualitative data? To my opinion, the quotations do not add a lot to the manuscript. | In line with best practice of thematic analysis [ref 53], we grouped the qualitative data into central themes, which were maintaining a sense of normalcy and social connections; technologies as double-edged sword; desired features of technology. The quotations were provided as evidence from the data that illustrates key elements of the themes.  We also discussed how the qualitative findings complement the quantitative findings in the Discussion:  “Qualitative findings complemented quantitative findings by showing while mobile devices helped maintain a sense of normalcy, there were potential negative effects of using technologies (e.g., stress and information overload from seeing COVID-19 information on social media, guilt when seeing low activity levels), which might have impacted users’ motivation and continued use of mobile devices. Our participants highlighted the need for technologies to adapt to changing circumstances.” (lines 400-406) |
| **Minor comments**:  Please check Table 1 to ensure that all categories reach 100% | We used conventional rounding methods (i.e., round up if the 2^nd^ decimal was five or above, and down if it was four or less). As a result, the sum does not equate exactly to 100%.  We have a footnote to Table 1 to explain this:  “^b^Sums may not equate to 100% due to rounding” (lines 262-263). |
